# Roles of the calcified cartilage layer and its tissue engineering reconstruction in osteoarthritis treatment

**DOI:** 10.3389/fbioe.2022.911281

**Published:** 2022-08-31

**Authors:** Weiyang Wang, Ruixi Ye, Wenqing Xie, Yueyao Zhang, Senbo An, Yusheng Li, Yang Zhou

**Affiliations:** ^1^ Department of Orthopedics, Xiangya Hospital, Central South University, Changsha, China; ^2^ Xiangya School of Medicine, Central South University, Changsha, China; ^3^ National Clinical Research Center for Geriatric Disorders, Xiangya Hospital, Central South University, Changsha, China; ^4^ Department of Orthopedics, Shandong Provincial Hospital Affiliated to Shandong First Medical University, Jinan, China; ^5^ Department of Clinical Nursing, Xiangya Hospital, Central South University, Changsha, China

**Keywords:** calcified cartilage layer, tidemark, osteoarthritis, scaffolds, engineering reconstruction

## Abstract

Sandwiched between articular cartilage and subchondral bone, the calcified cartilage layer (CCL) takes on both biomechanical and biochemical functions in joint development and ordinary activities. The formation of CCL is not only unique in articular cartilage but can also be found in the chondro-osseous junction adjacent to the growth plate during adolescence. The formation of CCL is an active process under both cellular regulation and intercellular communication. Abnormal alterations of CCL can be indications of degenerative diseases including osteoarthritis. Owing to the limited self-repair capability of articular cartilage and core status of CCL in microenvironment maintenance, tissue engineering reconstruction of CCL in damaged cartilage can be of great significance. This review focuses on possible tissue engineering reconstruction methods targeting CCL for further OA treatment.

## Introduction

In the structure of joints, the calcified cartilage layer (CCL), which is located between articular cartilage and subchondral bone, is bounded by the upper tidemark and the lower cement line. Multiple studies have suggested that CCL is significant either in the embryonic development of joints or normal joint activities. CCL alteration would disrupt its conventional physiological functions such as mechanical loading transmission, diffusion of materials, and cartilage calcification. When in metabolic musculoskeletal disorders, such as osteoarthritis (OA), CCL is altered accompanied by abnormal disruption of articular cartilage and subchondral bone due to biomechanical and biochemical changes. Thus, it is not sufficient to repair either articular cartilage or subchondral bone in OA treatment, and CCL regeneration is also critical. Tissue engineering reconstruction is suggested as a promising OA intervention. In this review, we summarize the structure and physiological function of CCL, as well as the pathological alteration in OA. Finally, we explore possible tissue engineering reconstruction methods targeting CCL for further OA treatment.

## CCL and the tidemark

### Structure of CCL

Articular cartilage consists of four highly organized zones: superficial, middle (transitional), deep (radial), and calcified layers (Buckwalter and Mankin, 1998; Sophia Fox et al., 2009). The chondrocyte phenotype and extracellular matrix (ECM) structure differ in each layer (Burr, 2004). The chondro-osseous junctional region, CCL, is defined as the mineralized cartilage which lies between the hyaline cartilage bounded by the upper tidemark and subchondral bone bounded by the lower cement line (Clark, 1990). Compared with the cement line, the tidemark appears to have more significant biomechanical functions (Madry et al., 2010), so we would state the role of the tidemark in the following sections.

As a fundamental structure in bone physiology (Wang et al., 2009a), CCL consists of dispersed, hypertrophic chondrocytes within lacunae in the calcified matrix which is composed of type I collagen, sodium hyaluronate, and nanohydroxyapatite in varying proportions (Zhou et al., 2022). Zhang, Ying et al. reported that the percentage of the dry weight of type II collagen as an organic compound of CCL was 20.16% ± 0.96%, lower than that of the hyaline cartilage layer (61.39% ± 0.38%); the percentage of the dry weight of hydroxyapatite (HA) as an inorganic compound was 65.09% ± 2.31%, less than that of subchondral bone (85.78% ± 3.42%) (Zhang et al., 2012). Inside CCL, perpendicular chondrocyte-derived collagen type II fibers become structurally cemented to collagen type I osteoid deposited by osteoblasts (Hoemann et al., 2012). The Fourier transform infrared spectroscopic imaging (FTIR-I) analysis reveals that the collagen content undergoes a stepwise increase from cartilage to bone, while proteoglycan only culminates within CCL (Khanarian et al., 2014).

Mature articular cartilage is integrated with subchondral bone through an approximately 20–250-μm-thick CCL (Hoemann et al., 2012). In one study of normal human femoral condyles, Wang et al. (2009a) reported that the mean thickness of CCL was 104.162 ± 0.87 μm, and the cell density of CCL was 51.25 ± 21.26 cells/mm^2^, which was much lower than that of the hyaline cartilage (152.54 ± 35.77 cells/mm^2^). They also discovered two junctional interfaces of CCL, with the “ravine-engomphosis”-shaped upper interface and “comb-anchor”-shaped lower interface, indicating the structural integration of the cartilage.

### Development of CCL

During embryonic development, CCL formation begins with ossification, which is the process in which mesenchymal tissues are gradually replaced by osseous tissues (Burr, 2004). Ossification is an indispensable way to form mature bones, and it includes two types: intramembranous and endochondral. The endochondral ossification begins with mesenchymal tissue transforming into a cartilage intermediate, which is later replaced by the bone under calcification—the deposition of calcium in the osseous tissue (Nishimura et al., 2012).

The endochondral bone growth occurs at the metaphyseal side of the growth plate, the proliferative area where cartilage continues to proliferate and be replaced by the bone in the diaphysis. There are also two chondro-osseous junctions between the epiphysis and diaphysis growth plate cartilage. The epiphyseal growth plate cartilage–bone interface has the tidemark, while the metaphyseal growth plate cartilage–subchondral bone interface has not (Kazemi and Williams, 2021). This indicates that the formation of CCL and tidemark in articular cartilage can be a common physiological process as the growth plate in endochondral ossification.

**FIGURE 1:**
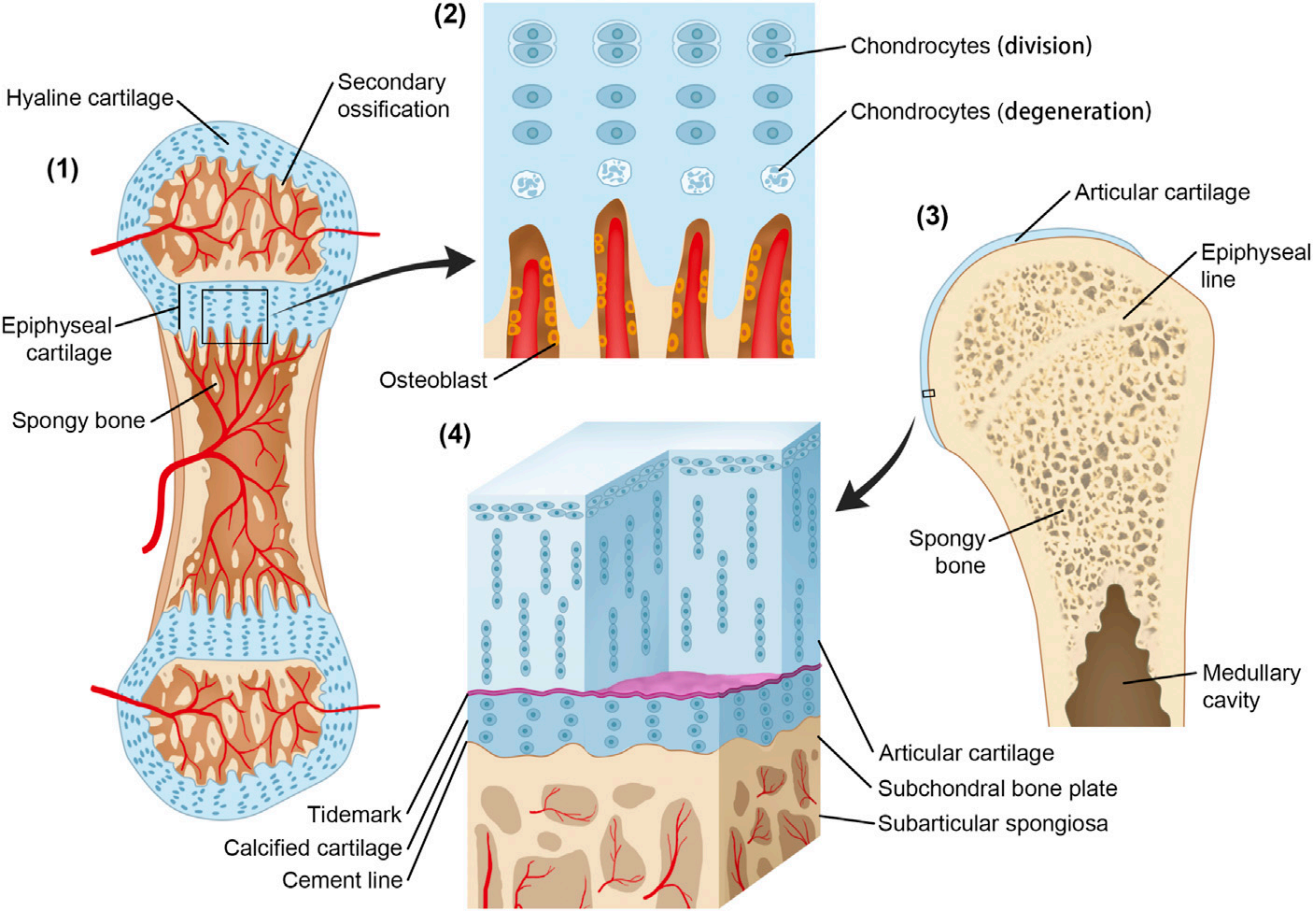
Origin and development of CCL and the growth plate. (1) Secondary ossification centers have formed. The initial structure of articular cartilage and growth plate comes from the same cartilaginous tissue. (2) Osteoblasts invade the lower boundary of the growth plate, replacing the cartilage with a bone; at the same rate, the growth plate enlarges through interstitial growth at the upper boundary by chondrocyte division and enlargement. The epiphysis is pushed away from the diaphysis, and the bone length increases. In contrast, the height of the calcified cartilage layer is maintained by the balance between tidemark advancement and bone transformation. (3) Following epiphyseal closure in adolescence, the calcified cartilage layer remains quiescent but can be reactivated in the ageing process and OA. (4) Microscopic three-dimensional structure of CCL.

According to the study by Müller-Gerbl et al. (1987), the height of CCL occupies a relatively constant proportion of articular cartilage, and it is achieved by the balance between the progression of the tidemark into noncalcified cartilage and changing into a bone by vascular invasion and bony remodeling (Oegema et al., 1997). This phenomenon can be associated with bone growth by the function of the growth plate (Figure 1). During its formation, the cells that form CCL have properties similar to the cells of the growth plate. At maturity, the rate of osteoblast activity exceeds that of epiphyseal cartilage enlargement, and epiphyseal closure occurs. Thus, growth plate cartilage disappears and is replaced by a distinct epiphyseal line. The two aforementioned events take place sequentially along the timeline. In adults, calcified cartilage remains quiescent but can be reactivated both with the aging process and pathological process including OA (Oegema et al., 1997).

### Tidemark

The tidemark, the junction of mature calcified and uncalcified articular cartilage, was first reported by H. T. Fawns and J. W. Landells in 1953 (Fawns and Landells, 1953). It is defined as a clear hematoxyphil line up to 10 μm thickness from a photomicrographic view (Gannon and Sokoloff, 1999). Nevertheless, it is not only simply an undulating line across a joint but also a complicated three-dimensional structure that follows uncalcified prolongations, penetrates the calcified cartilage, and impinges on subjacent bone or marrow spaces (Lyons et al., 2006). Since articular cartilage is an avascular, alymphatic, and aneural connective tissue, this prolongation could probably provide a route for both nourishment and interaction within the chondro-osseous junction.

The mature mineralization front is delineated by a thin approximately 5 μm undulating tidemark structure that forms at the base of the articular cartilage. Under static compressive loading, articular cartilage distortion could come to an abrupt end at the tidemark. It is said that a tidemark can act as a tethering mechanism for collagen fibrils in unmineralized cartilage (Huber et al., 2000), and alternations of orientation and packing density of the collagen framework in the tidemark region could be observed under load-bearing conditions (Broom and Poole, 1982). Orientation alternations of collagen fibers could have stress buffer effects, and it can be concluded that the tidemark has the function of stress transmission and corporation from compliant articular cartilage to rigid calcified cartilage.

Chen et al. (2011) reported that the tidemark appeared as a dense wavy line at the interface between calcified and hypertrophic layers of the condylar cartilage. They also found that thickness and distribution of the tidemark are loading-related. They showed a highly wavy tidemark surface in the load-bearing areas and a relatively flat and smooth surface in the non-load-bearing areas (Clark, 1990). Furthermore, the thickness of the tidemark was significantly higher in load-bearing areas than in non-load-bearing areas. Tidemark duplication represents the progression or reactivation of CCL. Although mostly identified in OA, this can be a common phenomenon in the normal articulation of all species (Oegema et al., 1997).

## Physiological roles of CCL

### Force transmission

From the traditional view, the mechanical transition from calcified cartilage to the subchondral bone undergoes a continuum of increasing stiffness (Hargrave-Thomas et al., 2015). Once the stiffness is discontinuous, shear stress will occur. This discontinuity exists between the cartilage and subchondral bone. As a transition layer, CCL can minimize the shear stress (Radin and Rose, 1986). Sandwiched between cartilage and subchondral bone, CCL shows transitional mechanical properties by stress facilitation (Broom and Poole, 1982). It is reported that calcified cartilage is 10–100 times more rigid than the cartilage but 10 times softer than the bone (Mente and Lewis, 1994), while alternation of this stiffness gradient is closely related to early degenerative changes in articular cartilage (Hargrave-Thomas et al., 2015).

Specifically, the transmission of force may be achieved through the network of branching collagen fibrils in CCL (Redler et al., 1975). The vertical collagen fibers transmit and dissipate stress, while the tidemark and cement line expand the contact area; thus, CCL fixes uncalcified cartilage on subchondral bone tightly and diffuses the load effectively, which contributes to the load-bearing capabilities of the joint (Green et al., 1970; Oegema et al., 1997; Zizak et al., 2003).

Additionally, it is reported that the mineral content in the calcified cartilage area increases exponentially, which is even higher than that in the bone (Khanarian et al., 2014). Compared with calcified cartilage, the bone requires less mineral content to achieve a similar elastic modulus (Gupta et al., 2005). This may explain that the elastic modulus of calcified cartilage is equivalent to that of subchondral bone in other studies (Burr and Gallant, 2012). With such high stiffness, it can be explained that CCL is always the first fraction zone when sudden trauma occurs (Keinan-Adamsky et al., 2005). Moreover, under repetitive traumatic injuries, including overload-related osteochondrosis, the collapse of CCL is also observed (Zhang et al., 2012). Above all, we can conclude that tidemark and CCL play a significant part in force transmission and structural stabilization.

### Material diffusion

In addition to taking on the biomechanical coupling role between articular cartilage and subchondral bone, calcified cartilage also assists biochemical communication (Boushell et al., 2017). With a lower diffusion coefficient than the uncalcified region, CCL acts as a barrier that limits diffusion and restrains vascular invasion from the subchondral bone (Boushell et al., 2017). Also, the low permeability of CCL could help stabilize the hypoxic environment of articular cartilage. However, the least diffusion capacity of the pathway upward does not equal none. The study by K.P. Arkill demonstrated that calcified cartilage permits small solutes to transport, and subchondral circulation may contribute much to the nourishment of the deep cartilage region (Arkill and Winlove, 2008). In this way, articular cartilage may get nutrition supplements both from superficial synovial fluid and the subchondral area. Based on fluorescence loss induced by photobleaching (FLIP), it is revealed through electron microscopy that the calcified cartilage matrix contained uncalcified regions (22% volume proportion) that are either large patches or myriad small regions among mineral deposition, which may function as transport pathways (Pan et al., 2009). In addition, since osteochondral vascularity is an indicator of pathological changes, calcified cartilage can restrain vascular invasion from subchondral bone to normally avascular hyaline cartilage as a direct physical barrier (Walsh et al., 2007; Staines et al., 2013).

Recent studies have shown that there is a network of nanochannels in both the calcified cartilage and bone, which may contribute to the transport of ions and small molecules. These nanochannels have a significant impact on the crosstalk between the cartilage and bone (Pouran et al., 2021; Tang et al., 2022). Pouran et al. (2021) found a three-dimensional continuous transition of mineralization architecture from the noncalcified cartilage to the calcified cartilage. They identified that the nanopore structure varies gradually with a radius of 10.71 ± 6.45 nm in calcified cartilage to 39.1 ± 26.17 nm in the subchondral bone plate, which indicated that connectivity of nanopores in calcified cartilage is highly compromised compared to the subchondral bone plate. Tang et al. (2022) also revealed densely packed nanochannels smaller than bone canaliculi (≈10–50 nm diameter) within the calcified cartilage and bone extracellular matrices but absent in the cement line. These novel discoveries emphasize the transportive role of CCL and the existence of possible direct signal pathways between articular cartilage and subchondral bone.

### Mechanisms of cartilage calcification

Crystal deposition, governed by a combination of physicochemical, cellular, and matrix factors, plays an essential role in the calcification of the cartilage (Bullough and Jagannath, 1983). The accumulation of calcium phosphate precipitates both inside and outside chondrocytes leads to cartilage calcification (Boskey, 2002). Deriving from observations of a particular cellular activity in the calcification front of articular cartilage, this process is correlated with extracellular matrix vesicles (Bullough and Jagannath, 1983). Calcification of the growth plate has similar underlying mechanisms as that of articular cartilage. Anderson (1969) also indicated matrix vesicles of different sizes (estimated 300 A to estimated 1 micro) and shapes derived from cells that are identified in the epiphyseal cartilage matrix, which may contribute to the initiation of cartilage calcification. More specifically, matrix vesicles enriched with microRNAs may possess significant regulatory functions on growth plate chondrocytes (Asmussen et al., 2021). Moreover, matrix vesicles presented with phospholipids also appear to be involved in the initial production of calcium HA crystals through the formation of phospholipid:Ca:Pi complexes (Boyan et al., 1989). Some other factors also participate in this process. Poole et al. (1984) emphasized that chondrocalcin may play a vital role in cartilage calcification by its unique affinity for HA. The inhibitory R2 fraction separated from protein–polysaccharide complexes are found to be degraded in the transient calcification zone, which may undergo the regulation of endochondral calcification (Pita et al., 1970). Regional cellular interactions of chondrocytes also regulate cell mineralization and matrix organization, and it is suggested that suppression of cartilage calcification under chondrocyte interaction is mediated by PTHrP (Jiang et al., 2008).

Hypertrophic chondrocytes derived from bone marrow mesenchymal stem cells (BMSCs) synthesized type X collagen and calcified the extracellular matrix around them (Yang et al., 2018). In the intermediate calcified process during endochondral ossification, along with the calcification of the matrix composed of type II collagen, type X correlates with type II collagen and may prevent the initial calcification of these fibrils (Poole et al., 1989; Kirsch and Wuthier, 1994).

## CCL alteration and OA progression

### CCL and OA

OA is a chronic joint degenerative disease with unbalanced bone homeostasis. In the progression of OA, uncalcified cartilage, CCL, and subchondral bone together form one functional unit as a whole. Subchondral bone is commonly divided into the subchondral bone plate and subchondral cancellous bone and undertakes primary pathological changes during early OA stages. Secondary articular cartilage alternations, including fibrillations, are always an indication of the severity and late OA stages.

**FIGURE 2:**
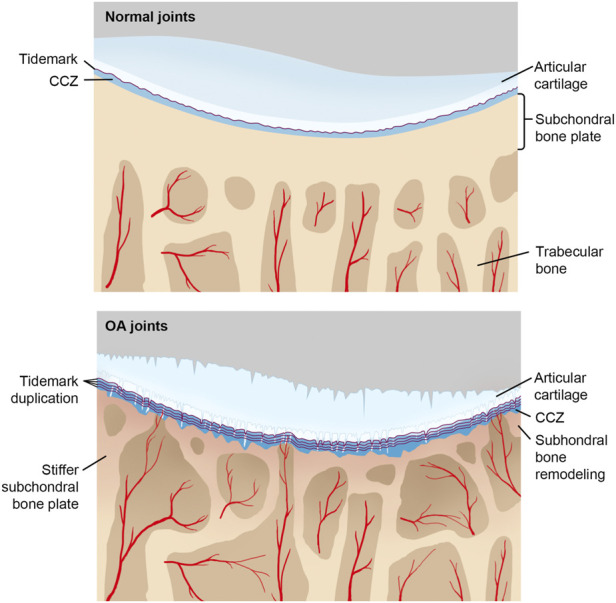
CCL in normal and OA joints with the progression of OA, tidemark advances, and calcified cartilage layer gets thicker, while noncalcified articular cartilage attenuates. Under repetitive stress, microcracks in the calcified cartilage layer take place, which promotes vascular invasion and associated repairing mechanisms. Moreover, sclerosis of both the subchondral plate and calcified cartilage aggravates too.

During early OA stages, constant remodeling of subchondral bone accomplished by a flourish of vascularity and active metabolism occurs (Aho et al., 2017). Meanwhile, microcracks in CCL take on both adaptive and repairing mechanisms to maintain cartilage integrity by recruiting osteocytes in the subchondral bone plate and initiating focal remodeling (Goldring, 2012; Zarka et al., 2019; Kaspiris et al., 2022). The increased remodeling rate undermines CCL and subchondral bone (Table 1). During the late stage of OA, subchondral cancellous bone continues to be osteopenic, while CCL and the subchondral bone plate thicken (Burr and Gallant, 2012). As OA progresses, tidemark duplication occurs, followed by fibrillation and deterioration of articular cartilage (Figure 2).

**TABLE 1:** CCL and subchondral bone changes during early and late stages of OA.

	CCL	Subchondral bone plate	Subchondral cancellous bone	Overall remodeling condition
Early stage of OA	Thinner	Thinner	(The trabecular plate) Thinner and more rod-like	↑Remodeling + osteopenia
Late stage of OA	Thicken	Thicken	Osteopenic	↓Remodeling + sclerosis

Recently, extensive research on mechanisms of OA-associated cartilage calcification has been published. DLX5-ALPL-IBSP-ENPP1 signal axis (Jiang et al., 2022), peripheral inhibition of SNS activity (Rösch et al., 2022), and overexpression of *TUFT1* gene (Sliz et al., 2017) promote OA-specific cartilage calcification. β2-adrenoceptor deficiency results in increased CCL thickness in the murine model study (Rösch et al., 2021). Pharmaceutically, the long-term application of dexamethasone increases the calcium content of CCL in both murine model and OA patients, which can also predispose OA and accelerate OA progression (Chen et al., 2021). In the contrast, a list of molecules that perform chondroprotective roles through inhibition of cartilage calcification is also found, including ciliary protein intraflagellar transport protein 88 (IFT88) (Coveney et al., 2022), betaine (Yajun et al., 2021), gasotransmitter hydrogen sulfide (H_2_S) (Nasi et al., 2021), and Tougu Xiaotong capsule (Li et al., 2013). Phosphocitrate (PC) and its analogs can restrain endochondral ossification by suppressing the hypertrophic phenotype (Hartlev et al., 2018). Both OA meniscal cells and chondrocytes have calcifying potentials (Kiraly et al., 2017), and recruitment of osteoclasts contributes to CCL microcracks and subjacent bone loss (Bertuglia et al., 2016).

As for the composition of CCL during OA progression, both collagen and proteoglycan contents undergo a decline, while the calcium/phosphate ratio is unchanged (Fan et al., 2022). Also, stiffened collagen fibrils are found in the cartilage–bone interface (Wen et al., 2012). When it comes to material properties, it is common sense that stiffness is always positively related to the mineral content. According to the study of Finnilä et al. (2022), CCL associated with pathological tidemark duplication has more mineral content than surrounding CCL portions, and CCL itself has more mineral crystals than adjoining bones. However, CCL still has a lower modulus, i.e., less stiffness, no matter in early or late OA stages, were former for increased bone remodeling and the latter for impairment of osteoblast regulation of calcification (Burr and Gallant, 2012) (Table 2).

**TABLE 2:** Changes of CCL and subchondral bone in different levels in OA progression.

	CCL	Bone
Gross morphology	Enhanced vascularization, Imhof et al. (1999)	
Microstructure	Multiple tidemarks, Burr, (2004)	Subchondral sclerosis, Burr, (2004)
	Thinner/thicker CCL, Burr, (2004)	↑Number and size of natural holes, Burr and Gallant, (2012)
	Microcracks, Imhof et al. (1999)	Microcracks (cortical end plate), Imhof et al. (1999)
Porosity	↑	
Composition, Fan et al. (2022)	↓Collagen	NA
	↓Proteoglycan	
	↑Mineral	
Material properties	↓Stiffness, Burr and Gallant, (2012)	
Cellular properties	Hypertrophic chondrocytes, Hwang et al. (2008)	

The thickness of CCL combined with the roughness of the tidemark and cement line can also be used to predict the reversibility of OA. In mild OA, the thickness first increases and later decreases, which indicates a reversible sign of OA pathological change. However, in moderate OA, the thickness continually increases, which indicates irreversibility (Deng et al., 2016). In addition to gross morphological and microstructural changes of CCL, specific biomarkers of CCL including calcium-binding protein, cadherin-binding protein, and carbohydrate metabolism-related proteins can also reflect the severity of OA (Fan et al., 2022).

### CCL functions as bone–cartilage crosstalk in OA

Bone–cartilage crosstalk exists in both normal joints and OA joints. CCL was traditionally considered an impermeable structure until 1994. Milz and Putz, (1994) first put forward the existence of channels between the uncalcified cartilage and the subchondral region. In normal joints, the transport of measurable solutes across CCL was detected by Pan et al. (2012). The vascular plexus is distributed along the undulations of the cartilage–bone interface reaches and perforates CCL, and finally provides nourishment to the hyaline cartilage (Pan et al., 2012). As mentioned previously, the 3D structure of the uncalcified cartilage also penetrates CCL and makes direct contractions with subchondral bones.

In OA, the bone–cartilage crosstalk capacity is elevated through channels mentioned previously, combined with microcracks (Burr and Schaffler, 1997; Burr and Radin, 2003) and vascular flourishment (Imhof et al., 2000; Pan et al., 2012). Small solutes and signaling molecules with calcifying potentials or joint harmfulness, as well as osteoblasts, all can transverse upward through those possible pathways in CCL. Thus, CCL may contribute to OA pathogenesis through this role of bone–cartilage crosstalk. Knee loading is found to perform the role of suppressing the differentiation of osteoclasts from bone marrow-derived cells and subsequent bone resorption of osteoclasts from the subjacent bone through the crosstalk (Li et al., 2016).

The rigidity of calcified cartilage, cortical end plate, and subchondral bone determines that microcracks and fissures after repetitive loading commonly occur in those sites. Since microinjuries may elicit a repairing mechanism, with the invasion of fibrovascular tissue, osteoclasts, and osteoblasts, the chondro-osseous junction undergoes remodeling, and blood vessels intrude. Subsequently, tidemarks duplicate (Imhof et al., 2000). The vascular canals in calcified cartilage promote fluid pressurization and affect hydraulic permeability of the subchondral bone plate. Increased interstitial fluid could affect the microenvironment in the chondro-osseous junction which leads to OA pathogenesis. According to Hwang et al. (2008), increased fluid exudation has deleterious consequences for cartilage and correlates with stages of OA. Thus, the increased hydraulic permeability secondary to angiogenesis enhances interstitial fluid flow, which also strengthens the bone–cartilage crosstalk.

## Strategies of CCL regeneration and the treatment for OA

Studies showed impaired CCL along with cartilage and subchondral bone defects in multiple bone disorders, including trauma, aging, and OA (Huey et al., 2012). The osteochondral structures, such as cartilage, have limited self-repair ability, which may lead to disease progression and eventually cause disabilities without effective treatments (Chen et al., 2009). The pathological changes of OA are featured with damaged cartilage and subchondral bone forming disturbed fissure and chondro-osseous junctions. Considering the physiological role of preventing cartilage vascularization and mineralization, the regeneration of the calcified layer may be critical for osteochondral defect restoration and OA treatment (Yang et al., 2018). In the following parts, we introduce the strategies of CCL regeneration (shown in Table 3) and its potential role in OA treatment.

**TABLE 3:** CCL regeneration strategies.

	Strategy
Conventional strategies	Subchondral drilling
	Microfracture
Allograft implantation	Autologous chondrocyte implantation
	Matrix-associated ACI
	Autologous and allogeneic osteochondral transplantation
OTE and CTE cell-based strategies	Digestion reagent-based strategies
	Chemotactic agent-based strategies
	Biomaterial-based strategies
OTE and CTE scaffold-based strategies	(Chemically modified) Scaffold (mostly hydrogels)
	Multi-phased layer scaffold
	Growth factor-induced BMSCs/scaffold
	Novel designed scaffold (3D printing)

OTE, osteochondral tissue engineering; CTE, cartilage tissue engineering.

### Conventional CCL regeneration strategies

Conventional strategies treating osteochondral injury, including subchondral drilling, microfracture, autologous chondrocyte implantation (ACI) and matrix-associated ACI (MACI), and autologous and allogeneic osteochondral transplantation, remain unsatisfying. Subchondral drilling and microfracture are beneficial for cartilage defect restoration through increasing osteochondral vascular supply. The limitation of these processes is that the fibrocartilage, which is functionally inferior to the cartilage, would be gradually formed instead of the cartilage in the injured sites (Beiser and Kanat, 1990; Sledge, 2001; Gobbi et al., 2020). ACI and MACI are reported to be applied clinically in articular cartilage regeneration with satisfactory outcomes (Gobbi et al., 2009; Kon et al., 2009; Saris et al., 2014). However, drawbacks such as chondrocyte source shortage, periosteal hypertrophy and ablation, insufficient regenerative cartilage, and graft delamination are repeatedly reported in ACI or MACI (Harris et al., 2011), as well as the low effectiveness for aged patients (Yang et al., 2017). Autologous and allogeneic osteochondral transplantation plays a vital role in massive osteochondral reconstruction and shows a good prognosis (Czitrom et al., 1986). However, the defective graft sources restrict its application, such as donor site morbidity of autografts, immune rejection, and disease spread of allografts (Yang et al., 2017).

### Tissue engineering strategies

Recent studies suggested that osteochondral tissue engineering and cartilage tissue engineering (OTE and CTE) have a promising performance in addressing the aforementioned limitations (Yang et al., 2017) with two main approaches regarding osteochondral interface and full-thickness cartilage regeneration, including cell-based and scaffold-based strategies (Liu et al., 2021; Petrovova et al., 2021) (Table 4). The principle of tissue engineering repair is the integration of grafts with the surrounding cartilage and subchondral bone. Digestion reagents, chemotactic agents, and biomaterial-based approaches are applied for cartilage–cartilage integration. In addition, CCL regeneration is critical for stable and functional cartilage–bone integration (Boushell et al., 2017). Lack of CCL was reported to contribute to decreased interfacial shear strength between the bone and cartilage (Kandel et al., 2006). Studies focused on CCL regeneration showed us encouraging results; however, due to the complex composition and structure of CCL, its regeneration remains challenging (Yang et al., 2018).

**TABLE 4:** Methods of CCL regeneration.

Type of strategy	Researcher	Time	Experimental model	Material	Reference
Scaffold-based	Petrovova E	2021	Sheep	Porous acellular PHB/CHIT-based scaffold	Petrovova et al., 2021
	Liu M	2021	Rabbit	PLCL-based tri-layered fibrous membranes	Liu et al., 2021
	Cai H	2020	Rabbit	Injectable tissue-induced Col I hydrogel and BMSCs	Cai et al., 2020

	Kosik-Kozioł A	2019	*In vitro*	Alginate, gelatin MAM, and β-TCP particles and BMSCs	Kosik-Kozioł et al., 2019

	You F	2018	Mouse	Porous ALG/HAP hydrogel	You et al., 2018
	Yang J	2018	Rabbit	Ica-HA/Col hydrogel and BMSCs	Yang et al., 2018
	Li Z	2018	*In vitro*	PLGA, HA, and extracted bovine cartilage matrix	Li et al., 2018
	Khanarian NT	2012	*In vitro*	HA and alginate hydrogel	Khanarian et al., 2012
	Cheng HW	2011	*In vitro*	Collagen and BMSCs (BMSC-collagen microspheres)	Cheng et al., 2011
	Jiang J	2010	*In vitro*	Agarose hydrogel and PLGA/45S5 bioactive glass	Jiang et al., 2010
Cell-based	Allan KS	2007	*In vitro*	DZCs, porous CPP, and β-GP	Allan et al., 2007
	Kandel R	1999	*In vitro*	Interface-relevant DZCs, Col II, and mineralization media (β-GP, PEA, and ATP)	Kandel et al., 1999

(a) PHB/CHIT, polyhydroxybutyrate/chitosan; (b) PLCL, poly(L-lactide-co-caprolactone); (c) Col I, type I collagen; (d) BMSCs, bone marrow mesenchymal stem cells; (e) MAM, methacrylamide; (f) β-TCP, β-tricalcium phosphate; (g), ALG/HAP: alginate/hydroxyapatite; (h) Ica-HA/Col, icariin-conjugated hyaluronic acid/collagen; (i) PLGA, polylactic-co-glycolic acid; (j) HA, hydroxyapatite; (k) DZCs, deep zone chondrocytes; (l) CPP, calcium polyphosphate; (m) β-GP, β-glycerophosphate; (n) Col II, type II collagen; (o) PEA, phosphoethanolamine; (p) ATP, adenosine triphosphate.

The cell-based strategy had been applied in previous studies. Kandel et al. (1999) successfully formed calcified cartilage with interface-relevant deep zone chondrocytes (DZCs), type II collagen, and mineralization media. Allan et al. (2007) completed a similar attempt, suggesting that DZCs are a promising cell source for CCL formation.

The scaffold-based approach has proved superior to the cell-based strategy because it requires less chondrocytes, and the functional properties of each tissue type can be readily achieved (Boushell et al., 2017). The scaffold ordinarily consists of hydrogels of natural (polysaccharides and proteins) or synthetic polymers [polyethylene glycol (PEG), polyethylene glycol fumarate (OPF), and polylactic-co-glycolic acid (PLGA), etc.] or their hybrids due to the satisfactory biocompatibility, biodegradability, and bioactivity (Holland et al., 2005; Wang et al., 2010; Nguyen et al., 2012; Luo et al., 2018). The biological properties of the scaffold could be optimized through chemical modifications. The incorporation of inorganic particles such as phosphate and silicate could improve the mechanical stiffness, osteoconductivity, and osteoinductivity of hydrogels (Yang et al., 2017). You et al. (2018) found that HA in alginate hydrogel could stimulate the secretion of the calcified matrix in chondrocytes. Similarly, Khanarian et al. (2012) introduced a scaffold of HA and alginate hydrogel which was reported to promote the DZC-mediated formation of a calcified cartilage-like matrix. Jiang et al. (2010) produced a continuous structure containing calcified cartilage with a scaffold composed of agarose hydrogel and PLGA/45S5 bioactive glass composite microspheres. In addition, layered OPF hydrogels and fibrin/PLGA hydrogels also showed the ability for CCL reconstruction (Wang et al., 2010; Steinmetz et al., 2015).

A multi-phased graft can guide the simultaneous regeneration of bone, cartilage, and a calcified cartilage intermediate. The early generation monolayer or bilayer scaffolds lacking the “boundary layer structure” (which acts as natural CCL) had the problem of excessive growth of the cartilage down into subchondral bone in the repair of osteochondral defects (Galperin et al., 2013), while many studies introduced tri-layered scaffolds with natural CCL and suggested excellent outcomes (Huang et al., 2021). Heymer et al. (2009) introduced a tri-layered scaffold consisting of a hydrophobic interface that separates cartilage and bone portions that are comprised of collagen I fibers with incorporated hyaluronan or polylactic acid, respectively. Kon et al. (2009) developed an acellular tri-layered osteochondral scaffold that controls the spatial distribution of calcium phosphate and collagen to recapitulate the cartilage-to-bone transition.

The implantation of BMSCs in the scaffold was reported to be indispensable for the regeneration of the biomimetic calcified layer (Cheng et al., 2011). Growth factors, such as TGF-β1 or BMP-2, could induce cartilage differentiation of BMSCs in type I collagen hydrogel (Nöth et al., 2007), while controlled delivery of these factors temporally and spatially might influence the BMSCs/scaffold structure in biomimetic calcified layer reconstruction (Wang et al., 2009b), but the huge cost, instability, and immunogenicity of these factors limited their application in clinical treatments (Mitchell et al., 2021). However, it has been suggested that growth factors were not necessary for this process because the type I collagen hydrogel scaffold might provide a suitable microenvironment for chondrogenesis (Zheng et al., 2010). Cai et al. (2020) restored a complete calcified interface and reconstructed cartilage homeostasis with BMSCs and tissue-induced injectable type I collagen hydrogel scaffold. In particular, the anatomical structure and physiological function of the cartilage repaired by this method were similar to the surrounding natural tissues. In another study, Cheng et al. (2011) constructed a tri-layered scaffold containing the chondrogenic layer, osteogenic layer, and middle undifferentiated BMSC–collagen layer. Then, they successfully formed a continuous and complete CCL with this scaffold. Yang et al. (2018) reported that both osteogenic differentiation and chondrogenic differentiation of BMSCs could be effectively promoted by Ica-HA/Col hydrogel *in vitro*, as well as the synthesis of type X collagen, which is an important marker for the formation of the calcified layer. Moreover, the *in vivo* experiment on a rabbit model showed that this hydrogel might contribute to the reconstruction of osteochondral sites. Recently, novel strategies have been applied focusing on the scaffold. Three-dimensional bioprinting has significant advantages in dealing with complex shapes and structures, with great potential in the reconstruction of CCL (Sheehy et al., 2013). Kosik-Kozioł et al. (2019) developed a 3D bioprinting hydrogel model using alginate, gelatin methacrylamide, and β-tricalcium phosphate (β-TCP) particles as a bioink. They also found that this model could promote the formation of the calcified zone by modulating the differentiation of BMSCs. Similarly, with PLGA and β-TCP, Li et al. (2018) constructed calcified layers of their multilayer scaffold through low-temperature 3D bioprinting technology. However, some challenges remain to be addressed, such as the low resolution of the printed matter, which limits its printing precision, and long processing time, which may reduce cell viability (Yang et al., 2017).

Novel scaffold designs utilizing a compositional gradient eliminate the need to integrate distinct layers. Harley et al. (2010) reported a gradient scaffold using liquid-phase co-synthesis with an unmineralized collagen II-chondroitin-6-sulfate cartilage region and a mineralized collagen I-chondroitin-6-sulfate bone region. The solutions are interdiffused at the interface, which could lead to a gradual transition in the composition between cartilage and bone sites. The design of multiphase osteochondral scaffold III: fabrication of layered scaffolds with continuous interfaces (Salerno et al., 2012).

## Summary

This review summarized the physiological roles of CCL and the ongoing studies focusing on CCL regeneration. CCL is suggested to play a significant role in force transmission, maintaining the stability of the osteochondral structure, and assisting the biochemical communication between the bone and cartilage. With the progress of OA, the normal structure and function of calcified cartilage are disrupted, and cartilage vascularization and mineralization occur, which is inconducive to osteochondral defect restoration. Therefore, the morphological, biochemical, and proteomic changes of CCL can reflect the progression of OA. CCL may contribute to OA pathogenesis through the role of bone–cartilage crosstalk which can be facilitated by both cartilage damage and vascular invasion in OA. However, the specific role of CCL in the occurrence and development of OA and its mechanism are still not very clear and need further research.

The regeneration of CCL is particularly important for osteochondral defect restoration, but it is still challenging. Compared with traditional regenerative strategies, tissue engineering shows great advantages in biocompatibility, strategy flexibility, repair integrity, and stability. The scaffold-based strategies have made successful attempts in the reconstruction of calcified cartilage both *in vivo* and *in vitro*. Chemical modification, the incorporation of inorganic particles, and the application of 3D bioprinting technology have flexibly improved the property of tissue engineering scaffolds, showing great research prospects, but the current research mainly focused on *in vitro* and *in vivo* experiments in small animals and not yet available for human. Maybe the organ culture models in large animal or human tissues could offer a promising integrated modality and are expected to address this limitation (Boushell et al., 2017). In addition, further elucidation of the role of intercellular interactions (like matrix vesicles mentioned previously) in maintaining the homeostasis of the osteochondral microenvironment and the occurrence and development of OA will be conducive to the development of new treatments.
